# Pharmacokinetics and pharmacodynamics of medication in asphyxiated newborns during controlled hypothermia. The PharmaCool multicenter study

**DOI:** 10.1186/1471-2431-12-45

**Published:** 2012-05-22

**Authors:** Timo R de Haan, Yuma A Bijleveld, Johanna H van der Lee, Floris Groenendaal, Marcel PH van den Broek, Carin MA Rademaker, Henrica LM van Straaten, Mirjam M van Weissenbruch, Jeroen R Vermeulen, Peter H Dijk, Jeroen Dudink, Monique Rijken, Arno van Heijst, Koen P Dijkman, Danilo Gavilanes, Anton H van Kaam, Martin Offringa, Ron AA Mathôt

**Affiliations:** 1 Department of Neonatology, Emma Children’s Hospital, Academic Medical Center, Amsterdam, The Netherlands; 2 Department of Paediatric Clinical Epidemiology, Emma Children’s Hospital, Academic Medical Center, University of Amsterdam, Amsterdam, The Netherlands; 3 Department of Neonatology, Wilhelmina Children’s Hospital, University Medical Center Utrecht, Utrecht, The Netherlands; 4 Department of Neonatology, University Medical Center Groningen, Groningen, The Netherlands; 5 Department of Neonatology, Radboud University Nijmegen Medical Center, Nijmegen, The Netherlands; 6 Department of Neonatology, Maastricht University Medical Center, Maastricht, The Netherlands; 7 Department of Neonatology and Radiology, Erasmus MC-Sophia, Rotterdam, The Netherlands; 8 Department of Neonatology, Maxima Medical Center Veldhoven, Veldhoven, The Netherlands; 9 Department of Neonatology, Isala Clinics, Zwolle, the Netherlands; 10 Department of Neonatology, Leiden University Medical Center, Leiden, the Netherlands; 11 Department of Neonatology, VU University Medical Center, Amsterdam, The Netherlands; 12 Department of Hospital Pharmacy, Clinical Pharmacology Unit, Academic Medical Center, Amsterdam, The Netherlands; 13 Department of Pediatric Neurology, VU University Medical Center, Neuroscience Campus Amsterdam, Amsterdam, The Netherlands; 14 Department of Clinical Pharmacy, Wilhelmina Children’s Hospital, University Medical Center Utrecht, Utrecht, The Netherlands; 15 Academic Medical Center, Meibergdreef 9, 1100 DD, Amsterdam, the Netherlands

**Keywords:** Perinatal asphyxia, Therapeutic hypothermia, Pharmacokinetic research, Drug monitoring, Evidence based, Drug dosing, Guideline

## Abstract

**Background:**

In the Netherlands, perinatal asphyxia (severe perinatal oxygen shortage) necessitating newborn resuscitation occurs in at least 200 of the 180–185.000 newly born infants per year. International randomized controlled trials have demonstrated an improved neurological outcome with therapeutic hypothermia. During hypothermia neonates receive sedative, analgesic, anti-epileptic and antibiotic drugs. So far little information is available how the pharmacokinetics (PK) and pharmacodynamics (PD) of these drugs are influenced by post resuscitation multi organ failure and the metabolic effects of the cooling treatment itself. As a result, evidence based dosing guidelines are lacking. This multicenter observational cohort study was designed to answer the question how hypothermia influences the distribution, metabolism and elimination of commonly used drugs in neonatal intensive care.

**Methods/Design:**

Multicenter cohort study. All term neonates treated with hypothermia for Hypoxic Ischemic Encephalopathy (HIE) resulting from perinatal asphyxia in all ten Dutch Neonatal Intensive Care Units (NICUs) will be eligible for this study. During hypothermia and rewarming blood samples will be taken from indwelling catheters to investigate blood concentrations of several antibiotics, analgesics, sedatives and anti-epileptic drugs. For each individual drug the population PK will be characterized using Nonlinear Mixed Effects Modelling (NONMEM). It will be investigated how clearance and volume of distribution are influenced by hypothermia also taking maturation of neonate into account. Similarly, integrated PK-PD models will be developed relating the time course of drug concentration to pharmacodynamic parameters such as successful seizure treatment; pain assessment and infection clearance.

**Discussion:**

On basis of the derived population PK-PD models dosing guidelines will be developed for the application of drugs during neonatal hypothermia treatment. The results of this study will lead to an evidence based drug treatment of hypothermic neonatal patients. Results will be published in a national web based evidence based paediatric formulary, peer reviewed journals and international paediatric drug references.

**Trial registration:**

NTR2529.

## Background

In the Netherlands, perinatal asphyxia (severe perinatal oxygen shortage) occurs in at least 200 out of 180–185.000 born infants/year. Term neonates experiencing a severe hypoxic-ischemic insult during birth may develop hypoxic ischemic encephalopathy (HIE) within hours. There is a high risk for long term neurological sequelae such as cerebral palsy, psychomotor retardation, and visual or auditory handicaps leading to long-term healthcare costs [1,2].

Cerebral hypoxia and ischemia result in several adverse biochemical events such as increased levels of excitatory neurotransmitters, excessive free radical production, an increase in intracellular calcium, and secretion of inflammatory mediators and messengers by microglial cells in the central nervous system initiating neuronal cell death [3-5].

Supportive treatment in the Neonatal Intensive Care Unit (NICU) comprises mechanical ventilation, cardiovascular support, and treatment of infections and seizures [6].

Animal research on controlled hypothermia following perinatal asphyxia showed a reduction in cerebral free radical and inflammatory damage [4,5]. Recent large randomized controlled trials and Meta analyses concerning the neuroprotective effects of hypothermia treatment in human asphyxiated neonates demonstrated a statistically significant and clinically important improvement of long term outcome [6-13]. Since 2008, all ten NICUs in the Netherlands have adopted controlled hypothermia as the standard of care for newborns suffering perinatal asphyxia.

Unfortunately, the potential benefits of therapeutic hypothermia could potentially be offset by decreased responsiveness to drug therapy and the occurrence of side effects due to the altered pharmacokinetics (PK) and pharmacodynamics (PD) during hypothermia [14,15]. Frequently used life-saving drugs in these newborns are sedatives, analgesics, antibiotics, and antiepileptic drugs (AED) and toxic side effects of these agents (e.g. cardiac arrhythmias from lidocaine; prolonged sedative effects from midazolam or morphine; nephrotoxicity –or ototoxicity for aminoglycosides) must be prevented.

There is evidence that the application of mild to moderate hypothermia decreases the systemic clearance of drugs metabolized by cytochrome P450 enzymes between approximately 7% and 22% per degree Celsius below 37°C [16].

The effects of hypothermia on drug metabolism have been investigated in humans but few studies concern drug metabolism in asphyxiated newborns. Sedatives and AEDs are important drugs used in the care of asphyxiated newborns. A decreased elimination rate constant (Ke) and clearance (CL) of midazolam was demonstrated during hypothermia in adult volunteers [17] but data on cooled neonatal patients are unknown. Recent findings suggest that phenytoin metabolism is inhibited by mild therapeutic hypothermia [18]. The administration of phenobarbital to newborns under whole body hypothermia has been reported to result in higher plasma concentrations when compared to normothermic newborns [19]. In non-cooled newborns an optimal lidocaine dosage schedule has been established [20], but the PK during cooling are unknown.

Analgesia is of major importance in neonatal intensive care as inadequate analgesia causes stress, counterproductive to the neuroprotective actions of hypothermia. On the other hand, toxic analgesic levels may cause prolonged sedative effects interfering with clinical neurological evaluations. Experimental studies demonstrated a 25% increase in plasma concentration of fentanyl at core body temperatures of 32°C [21]. Furthermore, Róka described elevated serum morphine concentrations and potentially toxic morphine levels in newborns with commonly used infusion rates of 10 μg/kg/hour during hypothermia [22].

Almost all newborns undergoing hypothermia treatment receive multiple antibiotic courses. The PK/PD properties of aminogycoside and glycopeptide antibiotics in these patients are largely unknown. The toxicity risks of gentamicin (nephro- and ototoxicity) in hypothermic newborns have only been evaluated in one study by Thorensen and colleagues [23]. A study in three adult cases concluded that induced hypothermia may result in impaired excretion of aminoglycosides [24]. The pharmacokinetics of penicillin and beta-lactam during hypothermia have not been studied yet.

Interestingly, other drugs do not seem to be influenced by cooling. A recent report concerning infants treated with the AED topiramate, a drug not licensed for use in neonates in the Netherlands at present, in a dose of 5 mg/kg/day, demonstrated drug concentrations within the reference range for the entire cooling treatment duration [25].

Altered pharmacokinetics during hypothermia may result in sub therapeutic as well as toxic drug concentrations Since many asphyxiated newborns will be exposed to controlled hypothermia evidence based guidelines for drug dosing (including loading and maintenance dose and dose interval) and therapeutic drug monitoring are urgently needed. The consequences of possible serious side effects or sub therapeutic dosing may have an unknown impact on survival or long term outcome of these infants.

In this Dutch multicenter study it will be investigated how controlled hypothermia influences the PK and PD time profiles of four major drug classes (i.e. analgesics, sedatives; antibiotic and antiepileptic drugs) used in the intensive care treatment of infants suffering perinatal asphyxia. Funding for this study has been received by the Dutch Government (ZonMw Grant number: 40-41500-98-9002).

## Methods/Design

A multicenter prospective cohort study in ten Dutch tertiary NICUs treating asphyxiated newborns with controlled hypothermia. Plasma concentrations of analgesic, antiepileptic, sedative and antibiotic drugs will be measured and used for population PK analysis to develop adjusted dosage regimens. Furthermore, the association of plasma drug concentrations with clinical effect will be investigated.

### *Project management, data management and safety monitoring*

A multicenter setup with a centralised study coordination and data management has been initiated. This will provide efficient use of existing resources and knowledge. All local principal investigators will receive GCP training according to European and international guidelines. The recent StaR Child Health Summit held on the 26th and 27th of October 2009 in Amsterdam initiated further international collaboration on defining GCP standards for PK/PD research in children. A dedicated clinical project manager will supervise logistics and data collection during the course of this study. A Clinical Research Associate (CRA) will conduct monitoring visits in all participating centres to monitor the adequacy of data acquisition, the completeness of clinical research forms (CRF) and the logistics and storage of samples. Laboratory visits to monitor sample processing and storage will be made every three months.

### *Serious adverse event policy*

The newborns in this study are severely ill and have a high risk for serious adverse events due to perinatal asphyxia or multiorgan failure. As no study related interventions will be performed besides blood sampling, only SAE in relation to this procedure will be reported to the steering committee and medical ethics committees of the participating centers. SAE as demise, severe multiorgan failure, pulmonary hypertension, treatment unresponsive seizures will be documented as related to the primary illness itself.

If SAEs occur due to unexpected toxic drug concentrations (e.g. severe cardiac symptoms during lidocaine therapy, prolonged unwanted sedation after midazolam therapy or renal toxicity in case of aminoglycoside treatment) these will be reported immediately to the steering committee which will directly communicate the nature and severity of the SAE to all participating centres.

### *Database and clinical research file design*

Each individual patient participating in this study will receive an anonimized personal study-identification number connected with an individual digital Case Record Form (CRF). Clinical and laboratory data (i.e. obstetric history, aetiology of asphyxia, Apgar scores, Thompson scores, birth weight, number of days in need of ventilatory support-or circulatory support, (co-)medication use, kidney and hepatic function tests, blood haematology and chemistry results, imaging results, sedation scores) are recorded in a web-based database, as well as long term outcome. Web-based CRFs are completed online by locally authorised and trained research staff. Participating centers will keep a written study file on site connecting study identification numbers with actual hospital identification numbers and patient files. The CRA fosters the collection of data and monitors all individual patient data at 3 month intervals.

For this study a web based research database was developed, which will serve as an instrument to evaluate the hypothermia treatment, to monitor treatment complications and and to assess long-term outcome of these critically ill infants. After termination of the study monitoring of outcome and complications of the hypothermia treatment will be continued using this database. Thus our multicenter national research group, and indirectly our patients, will continue to benefit from this unique infrastructure in future research.

### *Ethics committee approval*

The protocol for this observational study has been evaluated and approved by the local ethics committees of the following Dutch Organizations (all 10 NICU’s): The Academic Medical Centre of Amsterdam; The Erasmus Medical Centre of Rotterdam; The University Medical Centre of Utrecht, The Radboud University Nijmegen Medical Centre; The Maastricht University Medical Centre; the Maxima Medical Centre Veldhoven; The Isala Clinics, Zwolle; The Leiden University Medical Centre, The VU University Medical Centre of Amsterdam; The University Medical Center Groningen.

### Study protocol

#### Inclusion criteria

Any newborn:

· with a gestational age > 36 weeks and a birth weight > 3 kg;

· with Apgar Score at 5 min postnatal < 5;

· with continued resuscitation at 10 min postnatal;

· with 1 h postnatal blood gas analysis pH < 7.0 or base deficit > 16

· with clinical signs of moderate to severe encephalopathy (defined as a Thomson score of >7)

· who is undergoing neuroprotective treatment by controlled hypothermia < 6 h postnatal

#### Exclusion criteria

· congenital hepatic or renal pathology present (as this makes interpretation of PKPD results impossible);

· no central venous line or arterial bloodstream access for non-invasive blood sampling procedures

· no written parental consent to participate following informed consent interview.

#### Treatment protocol

Cooling: According to the national controlled hypothermia protocol, newborns with a history of perinatal asphyxia will be cooled to a core body temperature of 33.5°C for 72 h (starting within 6 h after birth). All participating centres have identical cooling equipment (Criticool Unit, MTRE Advanced Technologies Ltd, Israel). Following these 72 h infants will be re-warmed to a normal body temperature.

EEG-MONITORING All newborns will be continuously monitored for seizures by single or multiple lead aEEG (recorded on the Cerebral Function Monitor). To evaluate the severity of the encephalopathy aEEG background patterns will be interpreted according to current clinical evidence based definitions (Flat-trace, Continuous-Low-Voltage, Burst-Suppression, Discontinuous Normal Voltage and Continuous Normal Voltage). Seizure activity (status, recurrent seizures, incidental seizures) will be recorded [26]. All infants will also undergo a conventional full lead EEG during the cooling episode at 48 h following start of cooling [27].

The type, dosage and number of anti-epileptic drugs needed to control seizures will be recorded in the CRF.

#### Clinical encephalopathy scores and sedation

The clinical neurological status of all included newborns will be evaluated with the Thompson encephalopathy scores at study entry, during the cooling phase (daily, during 3 days) and after rewarming [28]. If encephalopathy scoring is not possible due to the administration of sedative drugs, this will be recorded in the CRF. From study entry on, pain and/or discomfort status of all newborns will be evaluated with the neonatal COMFORT score every 8 h. All changes in the dosing of sedative and analgesic drugs will be based on either an increase or decrease in the neonatal COMFORT score [29]. COMFORT scores will be recorded in the CRF.

#### Blood sampling – routine care

According to the national controlled hypothermia protocol all infants will have invasive blood pressure monitoring and central venous lines. All infants will have an indwelling urine catheter. All blood sampling procedures will be done on a daily basis according to the existing clinical protocols of controlled hypothermia. There will be no additional hematology or blood chemistry analysis for this study.

The current clinical protocol comprises:

1. Haemoglobin value, leukocytes (count and differential), thrombocyte count, clotting values (PT, APTT, fibrinogen).

2. Sodium, potassium, Creatinin, urea, liver enzymes (ASAT, ALAT, AF, bilirubin, Albumin, lactate, C-reactive protein.

3. Arterial blood gas analyses (twice daily)

4. Urine analyses: sodium; potassium; osmol; creatinin.

5. Blood cultures before start antibiotic treatment.

#### Blood sampling – pk/pd analysis

Additional blood samples will be taken for the evaluation of PK and PD. The sampling times are summarized in Table 1. No samples will be obtained on day 1 as a steady hypothermic state will not have been reached. The samples on day 2 and day 3 will be taken during the hypothermic state. On day 4 samples will be taken during rewarming and on day 5 during the steady normothermic state. In total a volume of 7.2 ml will be taken from the bloodstream by indwelling arterial catheters. If these lines are not functioning or cannot be placed, infants cannot participate in this study. As the average birth weight of a term newborn is 3.5 kg and the circulating blood volume is 80 ml/kg (total blood volume: 280 ml), 7.4 ml will correspond to 2.6% of the total circulating volume for the whole study. Blood samples for the evaluation of the PK-PD relationship will be taken together with clinically indicated blood sampling. The volume of blood drawn poses no clinical threat for the infant in terms of circulatory compromise or risk for anemia.

**Table 1:** Time schedule and volumes of PK –blood sampling

	Day 2	Day 3	Day 4	Day 5
07:30 (0.2 ml)	+	+		+
09:00 (0.2 ml)	+			+
11:00 (0.2 ml)	+			+
13:00 (1.2 ml)	+	+	+	+
15:00 (0.2 ml)	+			+
19:00 (0.2 ml)	+	+		+

#### Dosage of investigated drugs

All investigated drugs are given on clinical indications and will be administered by the intravenous route. Each patient will receive usual care during the whole study period in all participating centers. No dose adaptations will be made for study purposes. It is the goal of this study to evaluate the pharmacokinetic profile of currently used drugs in their current dose schedules. No new or experimental drugs will be evaluated in this observational study.

#### PK/PD analysis

PK and PD data will be analyzed during the normothermic and hypothermic state. Three groups of drugs will be investigated as, currently used in the participating NICUs:

Group I: Antibiotics: penicillin, amoxicillin, gentamicin, amikacin, vancomycin and ceftazidim

Group II: Analgesics: morphine and fentanyl.

Group III: Sedative and anti-epileptic drugs: midazolam, phenobarbital and lidocaine.

For each drug the dose, infusion rate, start time of infusion and end time of infusion are recorded in the CRF. Blood sampling times are recorded in the CRF as well. Samples are sent to the central study Pharmacy laboratory of the Academic Medical Center in Amsterdam. The samples will be labelled only with study numbers to guarantee the privacy of the patient.

State of the art liquid chromatography-mass spectrometric (LC-MS/MS) methods will be used to quantitatively measure drug concentrations in plasma. By using LC-MS, concentrations of drugs can be measured simultaneously in a small volume of plasma. The simultaneous quantification minimizes patient blood sampling and reduces study costs. Analyses of antibiotic plasma levels will be performed in the Pharmacy laboratory of the Academic Medical Center in Amsterdam and analyses of analgesic, AED and sedative plasma levels will be performed in the Pharmacy laboratory of the University Medical Center in Utrecht, the Netherlands. Analytical micro-assays has been developed and validated for the simultaneous analysis of phenobarbital, lidocaine (plus metabolite MEGX), morphine (plus 3- and 6-glucuronides) and midazolam (plus hydroxymidazolam and glucuronide) in 0.5 mL of serum. Simultaneous analysis of antibiotic drugs will be performed in 0.1 ml plasma.

#### PK/PD- data analysis

Nonlinear mixed-effects models (NONMEM) will be used to describe the time course of the drug concentration in plasma. Application of NONMEM allows average values for PK parameters as clearance and volume of distribution to be estimated as well as the inter- and intra-patient variability in these parameters. In these models it can be determined how hypothermia affects drug distribution, metabolism and excretion. Furthermore it can be evaluated whether specific patient factors (demographical, severity of asphyxia or end organ dysfunction/multi organ dysfunction) are related to changes in drug behaviour. Since PK models will be developed for neonates allometric scaling and maturation of PK parameters will be taken into account.

With a population PK model available different dosing schedules can be simulated in order to obtain adequate drug exposure during controlled hypothermia. The NONMEM-analyses will be performed in the Clinical Pharmacology Unit of the Hospital Pharmacy of the Academic Medical Center in Amsterdam (Prof. Dr R.A.A. Mathôt) and at the Department of Clinical Pharmacy of the University Medical Centre Utrecht in Utrecht.

#### Pharmacodynamic measures

Concentration effect relationships will be studied by NONMEM analyses as well. The following PD measures/endpoints will be used: successful seizure control (by anti-epileptic medication), adequate sedation or pain control by newborn stress scales (sedatives), adequate treatment of perinatal infection shown by negative repeat blood culture and correlation with MIC- values of cultured microorganisms. Also toxic side effects on end organs will be evaluated. (E.g. hearing damage caused by aminoglycoside medication evaluated by newborn hearing screening (ALGO-screening) or in case of abnormal ALGO; by brainstem auditory evoked potential investigation.).

#### Imaging studies & follow-up

A neonatal cerebral MRI will be performed in all patients as part of standard clinical care after the cooling procedure or if clinically indicated at an earlier stage. T1-and T2 weighted images and DWI (Diffusion Weighted Imaging) will be obtained in each patient. The MRI images will be evaluated centrally by skilled neonatologists and neuroradiologists. Long term follow-up of asphyxiated neonates is part of the standard care protocol of the Dutch Working Group of Neonatal follow-up. Long term outcome will be assessed by neurodevelopmental testing at the ages of 6 months, 1 and 2 years. When these long term outcomes become available, their association with perinatal pharmacological and clinical data will be investigated.

#### Sample size considerations

A formal power calculation cannot be performed as information is lacking. As a result our study has an explorative character. Nevertheless, as a rule of thumb data of at least 20 patients per investigated drug are considered to be sufficient to develop a population PK model.

#### Runtime

The total time for this study is three years. Inclusion will be stopped if the number of patients needed per drug (or medication group) has been reached. Each patient will followed-up until the age of 2 years according to the standard care protocol.

## Abbreviations

HIE = Hypoxic ischemic encephalopathy; AED = AntiEpileptic Drugs; Vd = Volume of distribution; CL = Clearance; CRA = Clinical research associate; CRF = Case record form: neonatal intensive care unit; SAE = Serious adverse event; LC-MS/MS = Liquid chromatography coupled with tandem mass spectrometry; MIC = Minimal inhibitory concentration; aEEG = Amplitude integrated electro encephalogram; NICU = Neonatal intensive care unit; DWI = Diffusion weighted imaging; PK = Pharmacokinetics; PD = Pharmacodynamics; NONMEM = Nonlinear mixed effects modelling.

## Competing interests

The authors declare that they have no competing interests.

## Authors’ contributions

T.R. de Haan, F. Groenendaal, C.M.A Rademaker, H.L.M. van Straaten, M.P.H. van den Broek Y.A. Bijleveld, J.H van der Lee, M.M. van Weissenbruch, R.J. Vermeulen, M Offringa and R.A.A. Mathôt were involved in drafting the concept and design of the study. All other authors were involved in the final consensus process of the protocol and contributed significantly to the final version. T.R. de Haan, Y.A. Bijleveld J.H van der Lee and R.A.A. Mathôt drafted the manuscript and all other authors read, edited and approved the final manuscript.

## Pre-publication history

The pre-publication history for this paper can be accessed here:

http://www.biomedcentral.com/1471-2431/12/45/prepub
